# Malignant melanoma and radiotherapy: past myths, excellent local control in 146 studied lesions at Georgetown University, and improving future management

**DOI:** 10.3389/fonc.2012.00167

**Published:** 2012-11-15

**Authors:** Pooya Jahanshahi, Nadim Nasr, Keith Unger, Ali Batouli, Gregory J. Gagnon

**Affiliations:** ^1^Virginia Commonwealth University School of MedicineRichmond, VA, USA; ^2^Department of Radiation Oncology, Virginia Hospital CenterArlington, VA, USA; ^3^Department of Radiation Medicine, Georgetown University HospitalWashington, DC, USA; ^4^Yale University School of MedicineNew Haven, CT, USA

**Keywords:** stereotactic radiosurgery, stereotactic body radiotherapy, malignant melanoma, linear quadratic models, gray units

## Abstract

**Introduction:** Once thought to be radioresistant, emerging cellular and clinical evidence now suggests melanoma can respond to large radiation doses per fraction. **Materials and Methods:** We conducted a retrospective study of all patients treated with stereotactic radiosurgery and stereotactic body radiotherapy at Georgetown University Hospital from May 2002 through November 2008 and studied the classic extrapolated total dose corrected for volume (ETD_vol_) model for predicting melanoma tumor response. Region-specific tumor outcomes were categorized by RECIST criteria and local control curves were estimated and analyzed when stratified by ETD_vol_ thresholds by use of the Kaplan–Meier method. **Results:** Follow-up information was available for 78 lesions (49 intracranial, 8 spinal, and 21 body) with mean follow-up period of 9.2 (range, 2–36) months. 1-year local control rates for intracranial, spinal, and body tumors were 84, 100, and 72%, respectively. Treatments in general were well-tolerated. Increased ETD_vol_ (*p* < 0.001) among intracranial sites resulted from larger (*p* < 0.001) doses per fraction combined with smaller (*p* < 0.001) tumor diameters. Intracranial 6-, 12-, and 24-month local control rates when treated above ETD_vol_ threshold of 230 Gy were all 90 vs. 89, 80, and 53% below this threshold. Body 6- and 12-month local control rates when treated above ETD_vol_ threshold of 100 Gy were 100 and 80% vs. 74 and 59% below this threshold. **Discussion: **By tailoring to melanoma’s unique radiobiology with large radiation doses per fraction, favorable local control was safely achieved. The ETD_vol_ model combines the important factor of dose per fraction in melanoma treatment with a volume correction factor to predict tumor response. Although limited sample size may have prevented reaching statistical significance for local control improvements using ETD_vol_ thresholds, optimal thresholds may exist to improve future tumor responses and further research is required.

## INTRODUCTION

Melanoma continues to be a significant cause of morbidity and mortality in the United States ranking as the fifth most common cancer in men and the seventh most common cancer in women (Jemal et al., 2010). Its incidence has rapidly increased over the past four decades, particularly among young adults (Reed et al., 2012), and although melanoma was once classically thought to be a radioresistant tumor based on cellular studies and clinical experience that utilized low-dose radiation (Barranco et al., 1971; Harwood and Cummings, 1981), it is now believed that melanoma cell lines have a wide range of sensitivity to radiation and can have favorable responses to large radiation doses per fraction (Stevens and McKay, 2006). The positive effects of large doses per fraction have now been confirmed by several clinical studies (Geara and Kang, 1996; Khan et al., 2011). Due to melanoma’s high tendency for locoregional recurrence and metastasis, radiation therapy is increasingly utilized in settings including postresection adjuvant therapy, palliative therapy, primary therapy, and additionally for unresectable tumors (Schild, 2009). Recent findings have shown promising results for melanoma tumors including for brain (Hara et al., 2009) and nodal (Burmeister et al., 2012) metastases and uveal melanoma (Modorati et al., 2009) among others. While still limited, the current data suggests malignant melanoma appears responsive to radiation when appropriate doses and fraction sizes are used.

Because large fraction sizes are a concern for late radiation complication risk, it is desirable to deliver radiation in a precise, highly conformal fashion with a sharp dose-gradient outside the tumor target, minimizing risk to proximal dose-limiting structures. These are characteristics of dose delivery using either stereotactic radiosurgery (SRS) or stereotactic body radiation therapy (SBRT) (Chang et al., 2003) making the assessment of these techniques critical in the development of improved melanoma radiation treatment. We studied outcomes in patients from our institution treated with SRS and SBRT. We further studied the utility of the extrapolated total dose corrected for volume (ETD_vol_) model as developed in the 1986 landmark Overgaard et al. (1986) study as a predictor of melanoma tumor response using our current findings.

## MATERIALS AND METHODS

### STUDY GROUP

This retrospective study was approved by the Institutional Review Board (IRB) of Georgetown University. 146 melanoma lesions treated with SRS and SBRT in 50 patients at Georgetown University from May 2002 through November 2008 were identified with mean patient age 56.6 years (range, 31–92 years). 143 lesions were metastatic and three were primary lesions located in the sella turcica, maxillary sinus, and orbit. Tumors were categorized by treatment site into intracranial, spinal, or body for all other sites. 89 tumors were intracranial, 19 were spinal, and 38 were located elsewhere in the body which included 11 lung and five neck lesions.

Chemotherapy was previously delivered to 26 patients. Intracranial lesions receiving prior treatment included four treated with gamma knife SRS, seven treated with CyberKnife SRS, 23 treated with whole brain external beam radiation, and four surgically resected. Spinal lesions receiving prior treatment included six treated with external beam radiation and 14 surgically resected. Body lesions receiving prior treatment included four treated with external beam radiation and five surgically resected.

### PROCEDURES

In our institution, SRS and SBRT are performed by the CyberKnife (Chang et al., 2003) and treatment of primary melanoma and melanoma metastases to intracranial, spinal, and other body sites including cutaneous, orbital, abdominal, oral, adrenal, pericardial, pulmonary, and sinus sites has been possible. Typically, SRS in the brain entails accurate delivery of a single or limited number of high-dose fractions. Hypofractionated SBRT can combine an ablative radiation dose with the use of modest fractionation to improve sparing of adjacent normal tissues.

### DATA ANALYSIS

Tumor outcomes were evaluated by serial CT, PET, and/or MRI scans and categorized into local failure, stable disease, partial response, or complete response by RECIST criteria (Eisenhauer et al., 2009). Local control was also reported and was defined as no local tumor progression. The Radiation Therapy Oncology Group (RTOG) toxicity scale was used. Last imaging dates and two-sided statistical tests were used in all calculations.

We also assessed the utility of the ETD_vol_ model as a guide for SRS and SBRT dose selection and for predicting tumor outcome. ETD_vol_ was given by Overgaard et al. (1986)

$${ETD}_{vol\,}=D\frac{d+2.5}{2.5}M^{-0.33}$$

where *D* was total dose (Gy), *d* was dose per fraction (Gy), and M was mean tumor diameter. We applied this equation to all 146 treated melanoma lesions. Odds ratios were calculated for region-specific local control rates as a function of ETD_vol_ above and below set threshold values.

The Kaplan–Meier method was used to estimate region-specific local control curves as well as to compare region-specific local control rates stratified by above or below ETD_vol_ thresholds using hazard ratios and log-rank tests. Time to local failure was censored by loss to follow-up and death due to non-study outcomes.

## RESULTS

### TREATMENT PARAMETERS

Total doses ranged from 750 to 5400 cGy (mean 2410 cGy) in 1–5 fractions. Mean target volume was 57.8 cm^3^. Mean prescription isodose line was 78.7% while the most commonly prescribed isodose line was 85%. Mean target volume covered by the isodose line was 96.5% with a mean ratio of max dose to prescribed dose of 1.27.

Mean tumor diameter was 1.6 cm for intracranial sites, 5.2 cm for spinal sites, and 5.4 cm for body sites. Mean dose per fraction was 1710 cGy for intracranial sites, 887 cGy for spinal sites, and 966 cGy for body sites. Using two-tailed *t*-tests, intracranial tumor dose per fraction was significantly larger than spinal (*p* < 0.001) and body tumors (*p* < 0.001), while intracranial tumor diameter was significantly smaller than spinal (*p* < 0.001) and body tumors (*p* < 0.001).

### TOXICITY

Using the RTOG toxicity scale, treatments in general were well-tolerated with the most common acute side effects being headache and nausea. These acute effects occurred exclusively in three patients treated for intracranial sites. At last follow-ups, no late toxicity was noted including myelitis and necrosis.

### CLINICAL OUTCOMES

**FIGURE 1:**
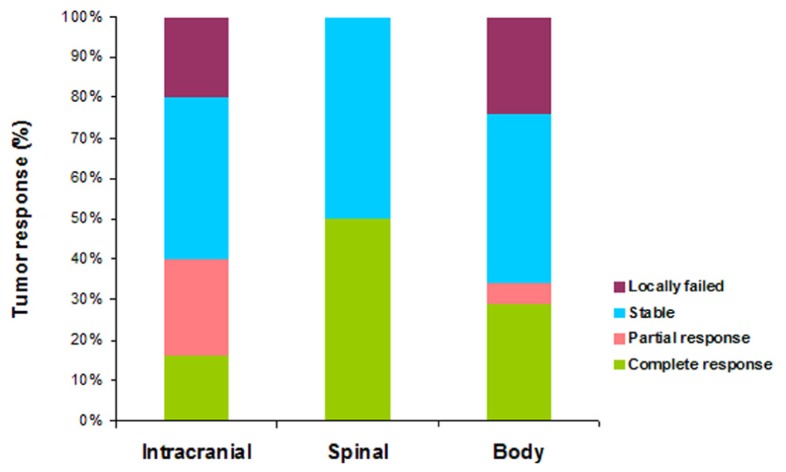
**Region-specific tumor outcomes categorized by RECIST criteria for intracranial (*n* = 49), spinal (*n* = 8), and body (*n* = 21) sites following SRS and SBRT**. Local control was achieved in 80, 100, and 76% of intracranial, spinal, and body sites, respectively.

Follow-up information for tumor outcomes was available for 78 lesions across 26 patients including 49 intracranial, eight spinal, and 21 body tumors which included five lung and four neck tumors. Mean follow-up period was 9.2 months (range, 2–36 months). Among intracranial lesions, eight (16%) showed complete response, 12 (24%) showed partial response, 19 (39%) were stable and ten (20%) failed locally after treatment. Among spinal lesions, four (50%) showed complete response and four (50%) were stable. Among body lesions, six (29%) showed complete response, one (5%) showed partial response, nine (43%) were stable, and five (24%) failed locally. All five lung tumors and two of three primary tumors were locally controlled with the sella turcica lesion being the sole primary melanoma lesion that locally failed. **Figure 1** illustrates tumor outcomes for intracranial, spinal, and body sites using RECIST criteria. To summarize, local control was achieved in 80% of intracranial tumors, 100% of spinal tumors, and 76% of body tumors.

**FIGURE 2:**
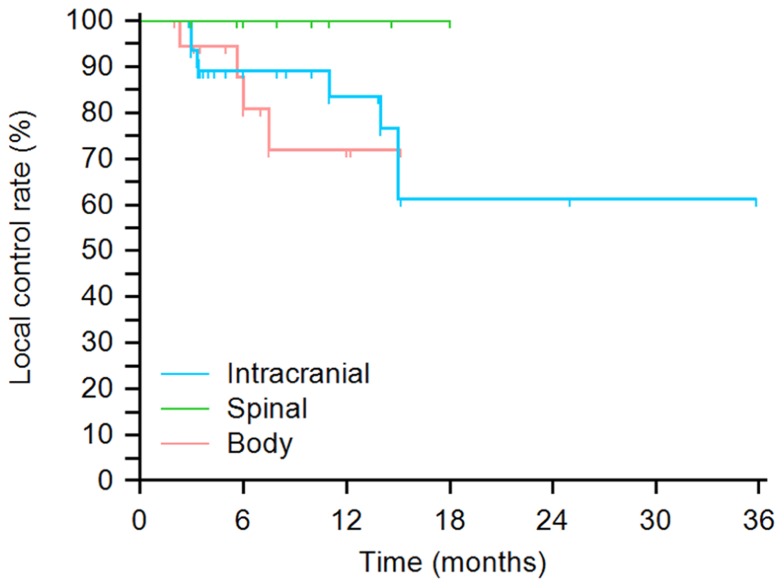
**Kaplan–Meier plot of probability of local tumor control by site**. No statistically significant local control differences were found between treatment sites (across all three sites log-rank *p* = 0.37, intracranial vs. spinal log-rank *p* = 0.22, intracranial vs. body log-rank *p* = 0.63, and spinal vs. body log-rank *p* = 0.16).

A Kaplan–Meier plot is displayed in **Figure 2** of time from treatment until local failure categorized by tumor region. No statistically significant local control differences were found between treatment sites. 6-, 12-, and 24-month local control rates for intracranial tumors were 89, 84, and 61%, respectively. 6-,12-, and 18-month local control rates for spinal sites were 100%. 6- and 12-month local control rates for body tumors were 81 and 72%, respectively. No statistically significant local control differences were found between treatment sites. Across all three sites log-rank *p* = 0.37, intracranial vs. spinal log-rank *p* = 0.22, intracranial vs. body log-rank *p* = 0.63, and spinal vs. body log-rank *p* = 0.16.

### ETD_vol_ THRESHOLD ANALYSIS

**FIGURE 3:**
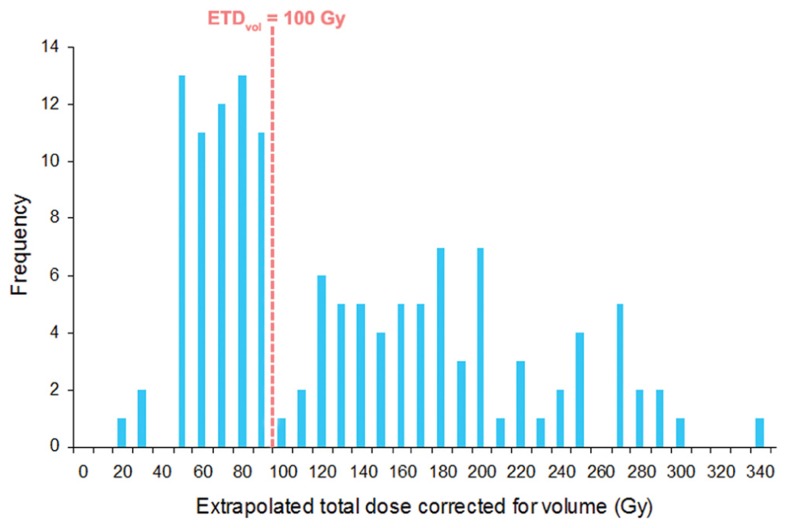
**Histogram of calculated ETD_vol_ for all 146 treated tumors**. Note the bimodal distribution with 62 of 74 (84%) tumors being intracranial tumors beyond the 100 Gy ETD_vol_ cut-off.

ETD_vol_ was calculated for all 146 lesions that received treatment (**Figure 3**). We noted a bimodal distribution of ETD_vol_ with a cut-off at 100 Gy. Across all tumor sites, mean ETD_vol_ was 126.7 Gy (range, 19.0–330.9 Gy). Mean ETD_vol_ was 159.7 (range, 44.3–283.4) for intracranial sites, 56.9 (range, 42.9–74.6) for spinal sites, and 96.1 (range, 21.0–245.8) for body sites. Two-tailed *t*-tests revealed mean intracranial ETD_vol_ to be significantly larger than mean spinal (*p* < 0.001) and body (*p* < 0.001) ETD_vol_. 62 of 74 (84%) tumor sites that received ETD_vol_ above the distribution cut-off of 100 Gy ETD_vol_ were intracranial sites. Of the 72 tumors that received less than 100 Gy, 27****(38%) were intracranial, 19 (26%) were spinal, and 26 (36%) were elsewhere in the body.

Tumor outcome as a function of ETD_vol_ could only be analyzed for intracranial and body tumors as there were no spinal failures. Using all available tumor outcomes, an ETD_vol_ threshold above 230 Gy for intracranial tumors yielded 90% (*n* = 10) local control with 40% (*n* = 4) complete response, 10% (*n* = 1) partial response, 40% (*n* = 4) stable, and 10% local failure compared to 79% (*n* = 39) local control below this threshold with 10% (*n* = 4) complete response, 31% (*n* = 12) partial response, 38% (*n* = 15) stable, and 21% (*n* = 8) local failure. Body tumors which received ETD_vol_ above a threshold of 100 Gy had 83% (*n* = 6) local control with 17% (*n* = 1) complete response, 0% partial response, 67% (*n* = 4) stable, and 17% (*n* = 1) local failure compared to 73% (*n* = 15) local control with 33% (*n* = 5) complete response, 7% (*n* = 1) partial response, 33% (*n* = 5) stable, and 27% (*n* = 4) local failure for tumors that received less than this threshold. Odds ratios for local intracranial and body tumor control when treated with doses above these thresholds were 2.70 and 1.82, respectively, however, no statistical significance was found (95% CI 0.30–24.28 and 0.16–20.71, respectively) possibly reflecting limited sample size.

Treatment groups above and below thresholds showed similar demographics with no significant differences in gender nor age (intracranial, gender proportion Fischer’s exact test *p* = 0.32, age two-tailed *t*-test *p* = 0.33, below threshold mean age 55.12 years and SD 14.47 years vs. above threshold mean age 61.75 years and SD 22.26 years; body, gender Fischer’s exact test *p* = 0.53, age two-tailed *t*-test *p* = 0.65, age below threshold mean 52.53 years and SD 17.47 years vs. above threshold mean age 56.33 years and SD 17.55 years).

**FIGURE 4:**
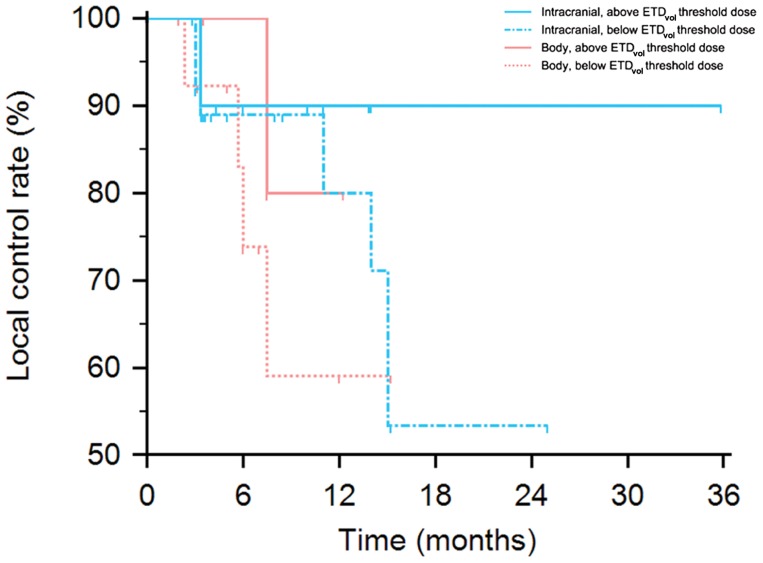
**Kaplan–Meier plot of probability of local control for intracranial and body tumors stratified by above or below ETD_**vol**_ thresholds of 230 Gy and 100 Gy, respectively**. Intracranial local control above vs. below threshold log-rank *p* = 0.36; body local control above vs. below threshold log-rank *p* = 0.34.

**Figure 4** displays a Kaplan–Meier plot of time from treatment until local failure comparing intracranial and body tumors when treated above and below respective ETD_vol_ thresholds of 230 Gy and 100 Gy. 6-, 12-, and 24-month local control rates for intracranial sites treated above ETD_vol_ threshold were all 90% while they were 89, 80, and 53%, respectively when treated below threshold ETD_vol_. 6- and 12-month local control rates for body sites treated above ETD_vol_ threshold were 100 and 80% while they were 74 and 59% for body sites treated below threshold ETD_vol_. However, statistical significance was not achieved possibly reflecting limited sample size (intracranial, hazard ratio [HR] 2.47, 95% CI 0.53–11.52, log-rank *p* = 0.36; body, HR 2.68, 95% CI 0.45–16.02, log-rank *p* = 0.34).

## DISCUSSION

We feel that it is feasible to deliver large doses of radiation in a highly conformal hypofractionated manner using SRS and SRBT to treat melanoma in metastatic and selected primary settings. Our local control (**Figures 1 and 2**) and toxicity were encouraging given the aggressive and morbid nature of the disease and compare favorably with other studies that have demonstrated a range of 68–89% local control of melanoma with SRS and SBRT (Hatiboglu et al., 2011; Bernard et al., 2012; Kelly et al., 2012).

Malignant melanoma’s historic classification among radioresistant tumors was, in part, related to intrinsic factors that were seen in *in-vitro* cell survival curves (Fertil and Malaise, 1981, 1987). Radiation cell survival curves for melanoma generally reveal a large shoulder which indicates a relatively high capacity to repair radiation damage (Bentzen et al., 1989). This implies that larger doses per fraction may be a better therapeutic strategy. Rofstad et al. (Rofstad, 1986; Rofstad et al., 1987) additionally found a role for extrinsic factors and tumor heterogeneity when they discovered radiation sensitivity of melanoma to vary within the same individual patient according to the site of biopsy. One such extrinsic factor for radiosensitivity is cellular oxygenation status at the time of radiation, and hypoxia is a prominent characteristic of melanomas. Re-oxygenation after fractionated radiation has been well-documented in these tumors, however, and the presence of this re-oxygenation may thus reduce the importance of hypoxia on tumor outcomes (McNally, 1980; Rofstad, 1989; Olsen and Rofstad, 1999).

The combination of radiobiologically important characteristics of hypoxia and a large shouldered cell-survival curve supports the use of hypofractionated radiation. The well-known risks of large dose fraction radiation on normal tissue argue for highly conformal dose delivery such as SRS and SBRT if this fraction scheme is to be used. Our favorable tumor outcomes and low toxicity utilizing these treatment modalities supported this line of rationale.

In a landmark study by Overgaard et al. (1986), dose per fraction was correlated with response in melanoma tumors while total dose, nominal standard dose, and treatment time were not. High doses per fraction yielded a significantly improved response (*p* < 0.001). The lack of influence of treatment time allowed a linear quadratic model analysis yielding an α/β ratio of 2.5 Gy. The authors were able to incorporate the α/β ratio and dose per fraction into a crude formula to estimate an isoeffect for different fractionation schedules. The authors improved this formula by correcting for tumor volume resulting in the ETD_vol_ formula. The authors calculated tumor volume by incorporating tumor diameter directly into the equation after empirical modifications found a best fit model. Although crude, this effectively removed the influence of tumor size and produced a significantly better prediction of response. The model combined the important factor of dose per fraction in melanoma treatment with a volume correction factor to achieve an accurately fitting dose–response model.

We similarly applied this equation to our current SRS and SBRT findings to evaluate its utility in determining optimal radiation schedules and improving future tumor outcomes. We first noted a bimodal distribution of ETD_vol_ with a cut-off at 100 Gy (**Figure 3**). Beyond the 100 Gy cut-off, tumors were found to be predominantly comprised of intracranial sites. Of the three variables which determine ETD_vol_, dose per fraction was significantly larger while tumor diameter was significantly smaller among intracranial sites. This had a combined effect to increase and right-shift intracranial ETD_vol_ resulting in the bimodal findings noted in **Figure 3**.

Spinal tumor ETD_vol_ thresholds could not be investigated due to complete local control of spinal lesions. Although limited sample size may have prevented reaching statistical significance for ETD_vol_ threshold analysis of intracranial and body sites, it appeared that ETD_vol_ thresholds may exist to improve tumor outcome. This would be in line with accumulating evidence suggesting high doses per fraction are necessary for optimal melanoma response. Treatment above a threshold ETD_vol_ of 230 Gy for intracranial and 100 Gy for body lesions resulted in higher local control rates when using all available tumor outcomes and higher 6-,12-, and 24-month local control rates.

Limitations of this study include treatment parameter differences resulting from catering to individual patients and lesions and relying on radiographic data from different imaging modalities for endpoint assessment. Future studies should also investigate symptom control as an endpoint for palliative cases and effects of previous and concurrent therapy on tumor outcomes.

Although this study lacked a control group, low-dose therapy has been classically found to be ineffective in controlling

melanoma tumors and mounting clinical studies (Geara and Kang, 1996; Stevens and McKay, 2006; Khan et al., 2011) suggest high dose per fraction to be the optimal method of treatment. Our excellent local control rates using high dose per fraction therapy appear to reaffirm this. In line with using high-dose therapy to improve tumor outcomes, we hypothesized that ETD_vol_ thresholds may exist above which tumor control is improved. While several studies report melanoma treatment doses, ETD_vol_, which importantly corrects dosages for tumor volume and could aid in optimizing dose schedules adaptable for future melanoma tumors, to the best of our knowledge has not been reported outside the original landmark Overgaard et al. (1986) study.

While there were no statistically significant local control improvements using ETD_vol_ thresholds, this may have been due to limited sample size as we did observe notable improvement above a 230 Gy threshold for intracranial lesions and 100 Gy for body lesions (overall: 90 vs. 75.7% in intracranial lesions and 83 vs. 73% in body lesions; intracranial 6-, 12-, and 24-month: 90, 90, and 90% vs. 89, 80, and 53%; body 6- and 12-month: 100 and 80% vs. 74 and 59%). Further research is needed to elucidate any statistically significant improved tumor responses that may exist using ETD_vol_ thresholds.

There appears to be little evidence of resistance to SRS and SBRT in the treatment of malignant melanoma. By tailoring to the unique radiobiology of melanoma with delivery of high doses per fraction, favorable rates of local control were safely achieved. Although SRS and SBRT as currently delivered can be effective in controlling these tumors, ETD_vol_ thresholds may exist to optimize radiation schedules and improve future melanoma tumor control. Data is still lacking before specific thresholds can be recommended and further research is required.

## Conflict of Interest Statement

The authors declare that the research was conducted in the absence of any commercial or financial relationships that could be construed as a potential conflict of interest
